# Slip detection for compliant robotic hands using inertial signals and deep learning

**DOI:** 10.3389/frobt.2025.1698591

**Published:** 2025-12-18

**Authors:** Miranda Cravetz, Purva Vyas, Cindy Grimm, Joseph R. Davidson

**Affiliations:** Collaborative Robotics and Intelligent Systems (CoRIS) Institute, Oregon State University, Corvallis, OR, United States

**Keywords:** compliant hand, slip detection, inertial measurement unit, grasping, contact-rich manipulation

## Abstract

When a passively compliant hand grasps an object, slip events are often accompanied by flexion or extension of the finger or finger joints. This paper investigates whether a combination of orientation change and slip-induced vibration at the fingertip, as sensed by an inertial measurement unit (IMU), can be used as a slip indicator. Using a tendon-driven hand, which achieves passive compliance through underactuation, we performed 195 manipulation trials involving both slip and non-slip conditions. We then labeled this data automatically using motion-tracking data, and trained a convolutional neural network (CNN) to detect the slip events. Our results show that slip can be successfully detected from IMU data, even in the presence of other disturbances. This remains the case when deploying the trained network on data from a different gripper performing a new manipulation task on a previously unseen object.

## 1 Introduction

Sliding between any two surfaces frequently results in frictional vibration (Ibrahim, 1994; Akay, 2002; Wang et al., 2020) – a fact which our bodies appear to exploit for our sense of slip. Evidence suggests that the fast acting mechanoreceptors in our skin, specifically the FA I receptors that sense vibrations under 40 Hz, play a dominant role in grip modulation (Macefield et al., 1996). This has motivated vibration-based slip detection methods for robotics, which have been studied since as early as 1989 (Howe and Cutkosky, 1989; Romano et al., 2011). However, such methods often struggle to distinguish between vibrations induced by slips (i.e., instances of sliding between the fingers and a grasped object) and those induced by non-slip disturbances such as contact events or environmental sliding (i.e., sliding between a stably grasped object and the environment) (Romeo and Zollo, 2020). Motivated by the observation that true slip events tend to be accompanied by a pose reconfiguration in passively compliant hands, in this paper we investigate whether joint flexion and extension in passively compliant hands can be used to discriminate between sliding-induced vibration caused by slip and sliding-induced vibration caused by environmental sliding.

**FIGURE 1:**
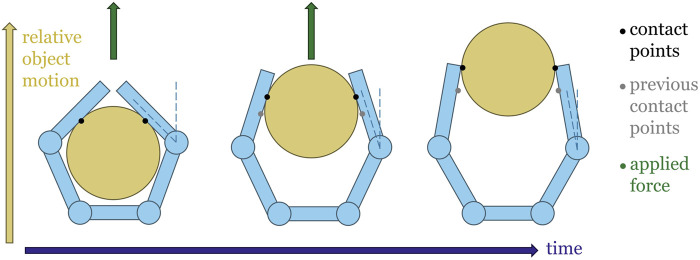
Slip occurs as a result of some applied force on the object. In compliant hands, which adapt their shape depending on the contact force at the fingers, this is accompanied by a change in joint angle. Section. 4.1.

Passive compliance is the ability of a structure to deflect in response to an experienced force without active actuation. Passively compliant grippers usually exhibit passive compliance about their primary joint axes, resulting in a situation where the final configuration of the gripper depends not only on actuation input, but also on the contact forces experienced by the fingers. The fingers of a passively compliant gripper may also be able to deflect in other directions or change their geometry. Passively compliant robotic grippers have several benefits when compared to their rigid counterparts. The shape-conforming ability of these devices allows them to grasp a wide variety of objects without complex control schemes (Shintake et al., 2018). Similarly, their ability to deflect in response to external forces makes them an increasingly popular choice for applications which require gentle manipulation, such as crop harvest (Navas et al., 2021) and medical applications (Tai et al., 2016). This same tendency to deflect in response to external disturbances results in a behavior in which the fingers of a compliant gripper tend to change configuration in response to the same physical disturbance that causes the slip between the grasped object and the gripper (Figure 1). Combining the slip-induced vibration response (as captured by an accelerometer) with this fingertip deflection (as captured by a gyroscope) may therefore serve as a slip indicator.

**FIGURE 2:**
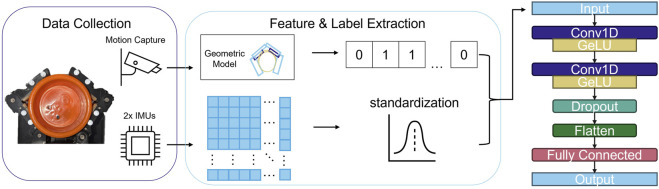
During the manipulation trials, the two finger gripper is placed in a motion tracking testbed. The accelerometer and gyroscope data from the fingertips is concatenated into feature matrices and preconditioned for machine learning. The motion tracking is used to reconstruct the scene geometry, which is in turn used to assign a binary slip label at each of a series of discrete timesteps. These feature matrices and binary labels are then fed into the CNN, the architecture of which is illustrated on the right.

For this study, we trained a convolutional neural network (CNN) to detect slip from data gathered from fingertip-mounted inertial measurement units (IMUs) (see Figure 2). We began by conducting a series of manipulation trials using a two-finger, tendon-driven hand. These manipulation trials included both true slip and environmental sliding conditions for five different grasped objects. We then automatically labeled this data as slip or non-slip using motion tracking data. We trained the CNN on a subset of this data and generated predictions on unseen examples using two classification thresholds. The network was able to achieve either an F1 score of 0.70 or a recall of 25.2% at a precision of 93.1% depending on the threshold. We additionally demonstrate the ability of the network to generalize to additional unseen objects and to a three-finger, tendon-driven gripper performing a forceful manipulation task. The specific contributions are as follows:

1. We provide an open-source dataset containing the motion tracking data, sensor data, kinematic data, and machine learning features and labels used in this paper. The dataset is available at https://zenodo.org/records/15886336.
2. We present a machine learning classifier that demonstrates the feasibility of using fingertip IMUs to detect slip in compliant hands.
3. We deploy the machine learning classifier on a different gripper design performing a practical manipulation task (fruit picking) on a previously unseen object.

## 2 Background

### 2.1 Discriminating slip from sliding

Slip sensing is a fundamental and much-studied aspect of robotic grasping. Romeo and Zollo (2020) provides a review of both current and classical slip detection methods. However, few papers address slip in the context of environmental contact, sliding, and robot motion. The most comprehensive work on discriminating between true slip and other disturbances appears to be Heyneman and Cutkosky (2016), which presented a method for classifying events as either slip (which Heyneman and Cutkosky call ‘object/hand’ slip) or environmental sliding (which they call ‘object/world’ slip, as in between the grasped object and something in the world around it). The authors achieved this classification by using dense arrays of tactile sensors, and were able to classify slip with a reported accuracy in the range of 49.38%–100%, depending on the sensor type and data pre-processing method. Later work examined the use of IMU arrays for the same purpose. Massalim and Kappassov (2019) calculated the cross-correlation of multiple IMU signals during slip events or environmental sliding and reported a difference in mean correlation between the two cases. However, that paper did not provide a complete slip detection method based on this statistical difference. Our work seeks to build on this work by creating and evaluating a predictive model.

### 2.2 Sensors for slip

Methods that use dense arrays of tactile sensors do show promise for contact-rich tasks. Such sensors provide enough information to reconstruct the contact area between the finger and the object and track its changes over time (Narita et al., 2020; Ruomin et al., 2021). Sensors commonly used for this purpose include the GelSight sensor (Yuan et al., 2017) and the Biotac sensor (SynTouch LLC, Los Angeles, California, United States). However, the cost and design constraints associated with these sensors can be problematic for some applications. For example, Gelsight and Biotac sensors are both based on fingertip deformation. If one of these deformation sensors is not isolated from the rest of a soft finger, the stretching and buckling of the finger material can be misinterpreted as contact at the sensor. There have been efforts to re-design these deformation-measuring sensors to be more suited to soft hands (Liu and Adelson, 2022), but the viability of emerging designs remains an active research area.

Another slip detection approach is to use individual or small arrays of force and pressure sensors. Recently, a few slip detection studies have been reported that take this direction. Both studies use grippers designed for robotic harvest of delicate crops, the type of task for which design constraints like size and flexibility may severely limit sensor choice. In Liu et al. (2024), researchers used a curvature and contact force sensor in each finger of a gripper to detect slip as weight was added to a grasped artificial fruit. This approach also incorporated vibration sensing by including the frequency of the changes in contact force measurements as part of the slip detection process. In Zhou et al. (2022), researchers used an arrangement of commercially available flexible pressure sensors embedded in a fin-ray gripper to detect sliding between the hand and an object. The researchers mounted the gripper palm-up and stably grasped an apple and apple tree leaf. They then slid the apple tree leaf relative to both the gripper and object, and used the resulting data to train a Long Short-Term Memory network. Our work seeks to extend these papers by examining a wider range of manipulation scenarios, which include gripper motion and sliding between object and environment, as well as a variety of grasped objects. Furthermore, we use IMUs as our slip sensor, which many researchers have already embedded in their soft and compliant hands, generally to reconstruct pose (Yu et al., 2023; Lin et al., 2024; Kieliba et al., 2018; Santaera et al., 2015).

## 3 Data collection

Our goal was to create a predictive model that could accurately identify slips during robotic manipulation, even in the presence of environmental sliding. We therefore aimed to create a dataset rich in examples of slip, sliding, and both combined. Motivated by a desire to make the results as applicable as possible, we performed a series of manipulation trials with a physical hand and objects. We designed four different manipulation scenarios (each combining environmental sliding and slip in different ways) for these trials, described in Section 3.1.1. Each manipulation type was performed several times using a range of grasped objects and initial grasps. This was done to improve generalization across factors such as surface friction, object shape, and grasp type on the model.

### 3.1 Manipulation trials

**FIGURE 3:**
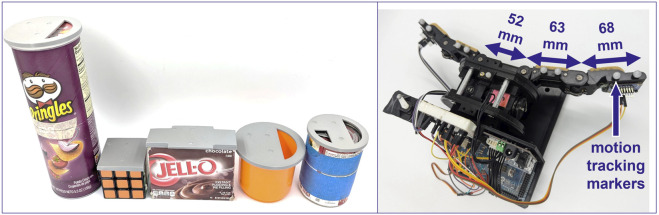
The object set *(left)* consists of a Pringles chips can, a Jello pudding box, a plastic cup, a Rubik’s cube, and a tomato soup can. Each object is equipped with a 3D printed retroreflective marker mounting cap. The custom two-finger gripper *(right)* is a modified Yale Openhand, equipped with fingertip IMUs, data collection hardware, and mounting points for motion tracking markers (gray spheres on fingers).

With our custom-made gripper (Section 3.2), we conducted a series of manipulation trials using five objects from the YCB Object Set (Calli et al., 2015): a Pringles chips can, a Jello pudding box, a plastic cup, a Rubik’s cube, and a tomato soup can. The objects were chosen based on their suitability for the gripper (e.g., size), rigidity, and ease of geometric representation (i.e., symmetry). For each object, we designed a close-fitting cap with indentations which we used to mount motion tracking markers in a fixed pattern (Figure 3, left).

For each trial, the gripper and a single object were placed in a motion tracking testbed with eight OptiTrack motion-capture cameras (NaturalPoint, Inc., Corvallis, Oregon, United States). We used the accompanying Motive software to log the data from these cameras. Markers were affixed to both the gripper and object in order to track their motion. The three dimensional position of each marker was recorded at a rate of 120 Hz using Motive (NaturalPoint, Inc., Corvallis, Oregon, United States). The marker positions and identities were sufficient to reconstruct the 6-dimensional pose of the gripper base, each individual link of the gripper fingers, and the object (see Section. 4.1).

**FIGURE 4:**
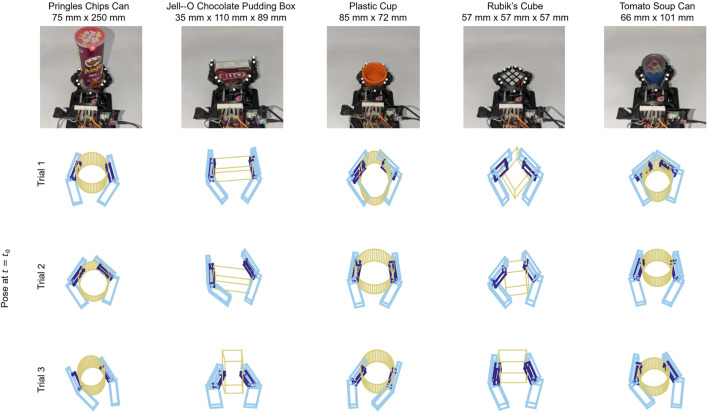
For each combination of grasped object and manipulation procedure, a variety of initial grasps were used. These included symmetrical and asymmetrical configurations, as well as both power and pinch grasps. Motion tracking data was used to reconstruct these grasps for label generation purposes. Three of these reconstructions are illustrated here for each object.

Each manipulation trial began with an experimenter actuating the two finger motors to achieve a stable grasp on the object. A variety of grasps were used for each object, including both power and pinch grasps. A subsample of example grasps can be seen in Figure 4. Once a stable grasp was achieved, the experimenter began a recording in Motive. The experimenter then ran a Python script that connects to the hand via serial connection and logs the data from the fingertip IMUs to a. csv file. This script also triggers a 0.5 s motion of the timing arm on the hand (See Section 3.2), allowing for time synchronization between the sensor data and the motion tracking data during postprocessing.

**FIGURE 5:**
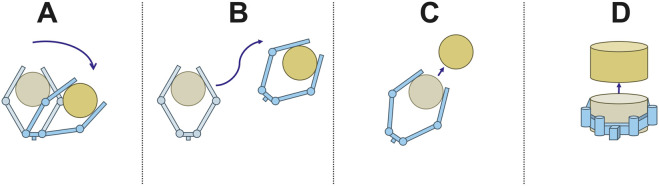
Illustrations of the four manipulation scenarios. In scenario **(A)**, the object shifts downward and to the right, and the fingers of the gripper reconfigure but stay in contact. In scenario **(B)**, the gripper rotates and translates while its configuration remains the same and the object remains stably grasped. In scenario **(C)**, the object is lost from the hand while moving in the plane. In scenario **(D)**, the object is lost while moving out of the plane.

Once a stable grasp was achieved, the experimenter then executed one of four procedures, representing different scenarios that may occur during autonomous, robotic manipulation of an object (see Fig. Figure 5 for an illustration). Two of the scenarios (labeled A and B below) represent manipulations where there is a significant amount of environmental sliding, but only a few minor slips. The grasp remains stable throughout most of the manipulation. In the case that the hand reconfigures, the object primarily rolls without slipping relative to the fingers. These two scenarios provide examples of environmental sliding both accompanied by—and not accompanied by—slip. Sliding between object and environment is frequently problematic for vibration-based slip detection methods, which is why we included it in our dataset.

The second two scenarios (labeled C and D below) represent failed manipulations where the object is completely lost from the hand. The only difference between the two scenarios is the direction of slip. In one case, it is in the plane of the hand, a direction in which the fingers are highly flexible. In the other case, it is out of the plane, a direction in which the fingers are comparatively stiff. A complete description of each scenario and how we performed them is as follows:

### 3.2 Manipulation scenarios

- Scenario A: The first scenario is a grasp reconfiguration caused by an external disturbance. This scenario involves motion of the gripper’s fingers and the grasped object relative to the base of the hand, but not necessarily sliding between the fingers and the object. To replicate this scenario, a researcher manually applied disturbances to the fingers of the hand while attempting to induce as little slip as possible, though some incidental slip did occur.
- Scenario B: In this case, which is counter to Scenario A, the configuration of the fingers and object remain static with respect to the base of the gripper, but the gripper and object move about in the plane, sliding along the surface on which they rest. To replicate this scenario, the researcher held the base of the hand and slid it on a surface. This included changing the orientation of the hand.
- Scenario C: In this procedure, the researcher manually pulled the object away from the base of the gripper in the plane of the fingers until the grasp failed.
- Scenario D: Here the researcher manually pulled the object out of the plane of the fingers, again until the grasp failed.

The gripper was secured to the bed of the motion tracking arena using bolts for all procedures except procedure B. Procedures A and B were conducted for 60 s per trial, and procedures C and D until the grasp failed (average duration of 12.5 s). We performed a total of 195,200 manipulation trials in this manner, divided evenly across object and procedure. Five trials had to be excluded due to data quality issues (missing or phantom markers, hardware communication failures, etc.) leaving 195 trials in the final dataset.

### 3.3 Hardware

We manufactured a two-finger, tendon-driven gripper for this study (Figure 3) based on the Yale Openhand (https://www.eng.yale.edu/grablab/openhand/) which is in turn based on the iRobot-Harvard-Yale (iHY) Hand (Odhner et al., 2014). We used heat set inserts to mount a commercially available circuit board (#3387, Adafruit Industries LLC, New York, NY) containing a single inertial measurement unit (LSM9DS1, STMicroelectronics NV, Geneva, Switzerland) to each distal fingertip. The accelerometer and gyroscope within the IMU were sampled at a rate of approximately 40 Hz using a multiplexer (PCA9546, Adafruit Industries LLC, New York, NY) to toggle between the two IMU breakout boards and an Arduino Mega to transfer the data over a serial connection to a laptop computer. We mounted 9 retroreflective markers to the gripper for motion tracking (see Figure 3), as well as a small motor-driven arm with an additional retroreflective marker. This arm was moved at the beginning of each grasping trial in order to temporally synchronize the motion tracking data with the data from the Arduino.

## 4 Automatic data labeling and dataset

We chose to approach slip detection as a binary classification problem, with the grasp labeled at each timestep as simply ”slipping” or ”not slipping”. These slip labels needed to be extracted from the raw motion tracking data, which we did by reconstructing the geometry and kinematics of the scene at each timestep and applying a first-order contact model. Furthermore, we construct two-dimensional feature matrices from the fingertip-mounted IMU data from which to predict slip.

### 4.1 Geometric reconstruction

**FIGURE 6:**
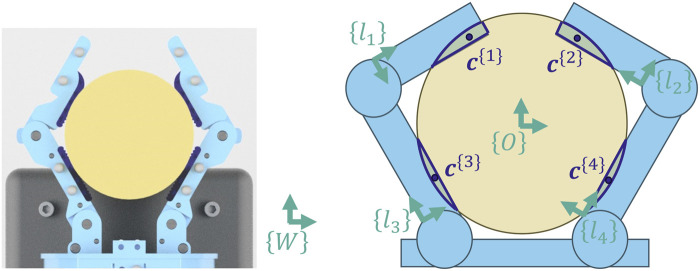
When an object is grasped, the elastomeric finger pads conform to the object (left). The reconstructed geometry is assumed to be rigid, which results in an overlap (right). The centroid of each overlapping region is treated as a contact point. The contact points are numbered based on the link of the gripper to which they belong.

To label the data, we begin by reconstructing the geometry of the hand and object at each timestep. We use Motive to track the three-dimensional positions of the markers on the hand and object. Let {W} be the world frame (defined by Motive). Using the measured locations of the motion tracking markers, we can reconstruct the six-dimensional pose of the object and each of the four finger links. More specifically, we attach a reference frame to each of these five bodies and encode the poses as homogeneous transforms between each frame and the world frame, {W} (Figure 6). We then model each body as a simple polyhedron using Geometry3D in Python (Minghao, 2020). The only exception is the distal finger links of the hand. Because they have an overhang that extends beyond the finger pad, they are each treated as a pair of rectangular prisms, arranged flush with each other. This results in three finger polyhedrons for each finger: a proximal finger link polyhedron, a distal link polyhedron representing the finger pad area, and a distal link polyhedron representing the overhang. The two fingers combined are therefore made up of six total polyhedrons.

For each time step we find contact regions between the fingers and object as follows. Since the objects and finger links are not perfectly rigid, there is a small deformation where the fingers contact the object. In our geometric model, this manifests as a small overlap between the non-deformable polyhedrons (Figure 6). Using Geometry3D, we calculate this overlapping intersection. Since the polyhedrons are all convex, this results in at most one contact region per finger polyhedron, for a maximum of six possible contact regions.

The contact region belongs equally to the two bodies in contact and can be thought of as two co-located regions: one on the object and one on the hand. Although these regions share the same location and shape, they do not necessarily share the same velocity. By looking at the difference between these velocities, we can determine whether slip is occurring.

### 4.2 Label generation

**FIGURE 7:**
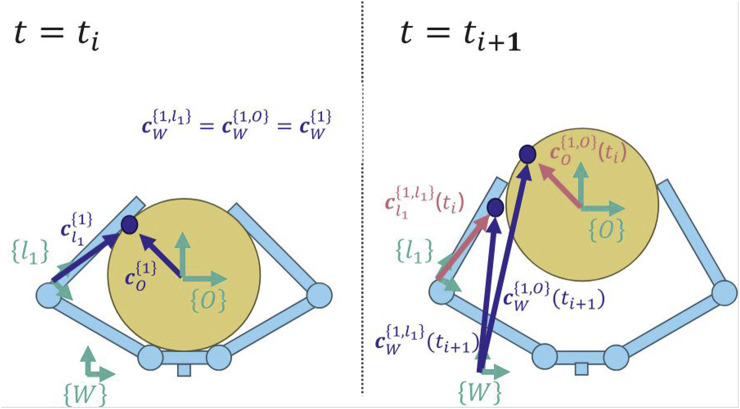
Each contact point is defined by a position along the link and a position on the object. At time $t_{i}$ , the contact point $c^{\left\{1\right\}}$ is shared by both link $l_{1}$ and the object (left). When time moves forward to $t_{i+1}$ , we can calculate the new location of the point on each respective body using their body-frame coordinates from the previous time (right). See Equations 2, 3 for more details.

Since the $j^{th}$ contact region is small, we reduce it to a point by finding the three-dimensional centroid of the region (Figure 6). We then find the location of this point in the local reference frame of the object ( $c_{W}^{\left\{j,O\right\}}$ ) and in the local reference frame of the relevant finger link ( $c_{W}^{\left\{j,l_{n}\right\}}$ ) (Figure 7). We next calculate the two velocities of the contact point. That is, for each contact point, we calculate both the velocity of contact point as it exists on the object ( $c_{W}^{\left\{j,O\right\}}$ ) and the velocity of the contact point as it exists on the link of the finger ( $c_{W}^{\left\{j,l_{n}\right\}}$ ). This allows us to apply the following slip condition:

$$\left|{\dot{c}}_{W}^{\left\{j,l_{n}\right\}}\left(t_{i}\right)-{\dot{c}}_{W}^{\left\{j,O\right\}}\left(t_{i}\right)\right|>\epsilon$$ (1)

That is, if the speed of the relative motion between the two bodies at the contact point is above the cutoff value, ϵ , the surfaces are considered to be sliding relative to each other. We neglect the possibility of pure contact breaking, since it is rare in practice for the contact point velocities to be perfectly parallel. This definition of slip corresponds to the common kinematic definition of sliding as any non-zero relative velocity at the contact point, but allows a margin around zero. Including this margin allows us to discard false events caused by noise, as well as, if desired, small sliding events that may not be task-relevant. If the condition is met for any of the contact points, then we label the example as a slip event. For this study, we used an ϵ value of 6 cm/s, which we chose experimentally. We found that the proportion of slip in the dataset was relatively stable for cutoff values between 4 cm/s and 8 cm/s, whereas cutoff values outside of this region resulted in dramatic class imbalances. We also found that models trained on labels that used a cutoff near the center of this region showed improved performance. Our chosen cutoff for this study results in a dataset that is approximately 40% slip (see Table 2).

We find ${\dot{c}}_{W}^{\left\{j,l_{n}\right\}}$ and ${\dot{c}}_{W}^{\left\{j,O\right\}}$ using a backwards-difference method over the point positions. Specifically, using the definitions in Table 1, we apply the following formula:

$${\dot{c}}_{W}^{\left\{j,l_{n}\right\}}\left(t_{i+1}\right)=\frac{1}{\Delta t}\left(T_{Wl_{n}}\left(t_{i+1}\right)c_{l_{n}}^{\left\{j,l_{n}\right\}}\left(t_{i}\right)-c_{W}^{\left\{j,l_{n}\right\}}\left(t_{i}\right)\right)$$ (2)

$${\dot{c}}_{W}^{\left\{j,O\right\}}\left(t_{i+1}\right)=\frac{1}{\Delta t}\left(T_{WO}\left(t_{i+1}\right)c_{O}^{\left\{j,O\right\}}\left(t_{i}\right)-c_{W}^{\left\{j,O\right\}}\left(t_{i}\right)\right)$$ (3)

**TABLE 1:** Definitions of terms.

{W}	World frame
{O}	Body frame of the grasped object
$\left\{l_{n}\right\}$	Body frame of the $n^{th}$ link
$c_{W}^{\left\{j,l_{n}\right\}}\in R^{3}$	Centroid j represented in world frame and attached to link n
$c_{W}^{\left\{j,O\right\}}\in R^{3}$	Centroid j represented in world frame and attached to the grasped object
$T_{Wb}\in SE\left(3\right)$	Transform representing body frame {b} in the world frame, e.g., $T_{WO}$ or $T_{Wl_{n}}$

The first product (i.e., left product) starts with the location of each contact point from the previous time step, as expressed in the frame of the associated link (Equation 1) and the frame of the object (Equation 2), then uses the present poses of the links $T_{Wl_{n}}\left(t_{i+1}\right)$ and object $T_{WO}\left(t_{i+1}\right)$ to project these locations forward in time and find the new location of these points in the world frame (see Figure 7). The difference between this new (present) position in the world frame and the previous position in the world frame is used as a displacement. We then divide by the elapsed time to calculate the velocity of the points. Finally, we smooth the velocities by applying a second-order Savitzky–Golay filter with a window length of 9 samples to the velocity in each axis.

### 4.3 Feature selection

**FIGURE 8:**
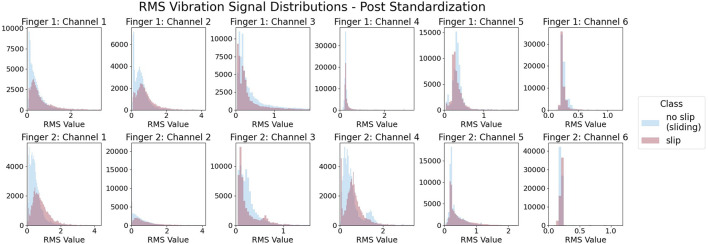
Within each IMU channel, both slip and non-slip samples show similar levels of vibration. Here, vibration is measured by root-mean-squared (RMS) activity of the standardized signals.

To create the features we downsample the motion data to a rate of 40 Hz. For each resulting timestep, we create a feature matrix from the previous 30 IMU samples from that point in time, equivalent to a 0.75 s window of data. Each of the two fingertip IMUs has three gyroscope and three accelerometer data channels, resulting in 12 total features per sample. This 12 by 30 feature matrix becomes associated with the slip condition at that point of time as a paired feature and label. We then remove any examples from the dataset where neither the hand nor object was in motion for the entire 0.75 s data window, as determined by the motion tracking data. Since the hand and object are in contact with the environment throughout the entirety of each trial, this removal process leaves only data containing environmental sliding. Because sliding is always present, the overall vibrational activity on the IMUs is comparable for both classes, as seen by Figure 8.

Finally, we divide the data by randomly placing 20% in a testing dataset. We then divide the remaining trials into a variety of train-validate pairs depending on the goal. For performance evaluation, we randomly divide the trials with a 6:2 ratio of training to validation data. To evaluate the ability of the network to generalize to unseen objects, we generate five additional pairs of training and validation data, using a leave-one-out technique. That is, we divide the data by moving all trials that were performed with a particular object to the validation set and use the remaining data for training. The final number of examples in each dataset are recorded in Table 2 and Table 3.

**TABLE 2:** The number of examples and class distribution in each category for the primary dataset.

Dataset (% slip)	Train	Validation	Test	Total
Examples	135,492 (41.5%)	46,004 (41.0%)	49,293 (38.3%)	230,789 (40.7%)

**TABLE 3:** The number of examples and class distribution in the leave-one-out datasets. These datasets contain the same examples as the combined training and validation data from Table 2.

Object	Train - object excluded	Validation - only this object (% slip)
Pringles can	140,609	40,887 (2.4%)
Pudding box	137,779	43,717 (34.5%)
Plastic cup	151,112	30,384 (80.1%)
Rubik’s cube	158,341	23,155 (65.0%)
Soup can	138,143	43,353 (43.2%)

## 5 Fruit picking dataset

**FIGURE 9:**
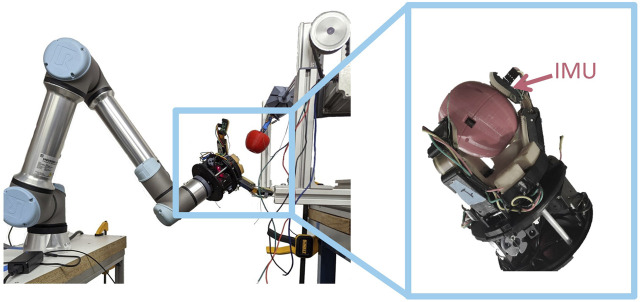
We completed 77 apple picks on our physical orchard proxy (*left*); each pick attempt was videotaped and then manually inspected to identify if and when slip occurred. Each distal link has an imu embedded under the fingerpad (*right*).

To test the applicability of our trained network to real-world robotic manipulation tasks, we gathered additional data from a robot performing a fruit-picking task. We first recorded video and sensor measurements from 77 pick attempts on our physical proxy orchard system described in Velasquez et al. (2022). In our previous work, we demonstrated that models trained on in-hand data from a fruit picking task using the proxy orchard perform comparably in the orchard to those trained on data collected directly from the orchard itself. These pick attempts were performed with our custom three-fingered end effector (Dischinger et al., 2021) attached to an industrial manipulator arm (UR5e, Universal Robots, Odense, Denmark) (see Figure 9). Each distal link of the end effector is equipped with a 6-axis inertial measurement unit (MPU6055), which was sampled at a rate of 70 Hz. For each pick attempt, we replicated the relative starting pose of the gripper with respect to the apple from one of our field trials (Velasquez et al., 2022), then added a small random offset to the position and orientation to create a unique pick attempt. The gripper then closed, and the robot pulled back a fixed distance along a path coincident with the vector normal to the palm. We reviewed timestamped video of the experiments and recorded the start and end time of any visually-observable slip events. These times were used to label the grasp at every experiment time as either slipping or static (i.e., no visual change in the pose of the apple with respect to the gripper). That is, times after the slip started and before it concluded are labeled as slip and all other times are labeled as static.

We formatted the fruit picking data in the style of the dataset on which our model was trained. We downsampled the IMU data to 40 Hz to match the sample rate used in our manipulation trials with the two finger gripper. Since the hand used in those trials had only two fingers, we constructed feature matrices for each possible pair of fingers on the three-finger gripper (fingers 1 & 2, 1 & 3, and 2 & 3). These were constructed using the same method described in Section 4. This resulted in three sets of feature matrices, one for each of the three finger pairs, and one set of shared labels. We pre-conditioned this data to have a mean of zero and standard deviation of one for each feature. A total of 1,179 examples (3.3% slip) were generated in this manner from across the 77 trials.

## 6 Models and experiments

We implemented a neural network architecture composed of two one-dimensional convolutional layers followed by a fully connected output layer. The convolutional layers have a stride of one over the dimension of the feature matrix that represents time. Each convolutional layer is followed by a Gaussian Error Linear Units (GELU) activation function. We apply dropout between the convolutional layers and the fully connected layer. This architecture is visualized in Figure 2.

### 6.1 Training and performance evaluation

We began by training and validating the model. First, using hyper parameter optimization, we determined the best:

- number of filters in each convolutional layer
- size of the filters in each convolutional layer
- dropout rate
- learning rate and learning rate decay
- weights for our weighted binary cross-entropy loss function
- weight decay for L2 regularization

Specifically, we used the Tree-Structured Parzen Estimators algorithm (Bergstra et al., 2011) in Hyperopt (Bergstra et al., 2013) to find a set of hyperparameters that maximized the F1 score of the model on the validation dataset (see Section 4). In the final model, the first convolutional layer applies 7 3 × 3 filters, the second convolutional layer applies 4 3 × 3 filters, and the dropout rate is set to 0.4. Training-related hyperparameters are available upon request.

During training, each example’s class is predicted using the argmax function over the output logits. During validation, a softmax function is applied instead and the class is predicted based on a confidence threshold for the positive (slip) class. During validation we also benchmark our algorithm against two classical methods: a k-nearest neighbors (KNN) classifier and support vector classification (SVC).

We evaluate the overall performance of our trained network on our reserved testing data, with the consideration of two use cases: a conservative use case where false positives are considered more detrimental than false negatives and a balanced performance use case where both precision and recall are similarly important. We evaluated the true positive rate and false positive rate at a variety of thresholds and used the resulting receiver operating characteristic (ROC) ROC curve to determine two thresholds for testing. The first threshold is the minimum threshold at which the network achieves 95% precision. This represents the conservative case. The second threshold is where the maximum F1 score is achieved, representing the balanced case.

### 6.2 Object generalization

As a way to assess the potential of the model to generalize to unseen objects, we went on to train five additional networks on the five leave-one-out training datasets described in Table 3. We kept the same network architecture and training procedure as above. We evaluate the performance of these five networks on the corresponding single-object validation datasets and compare this with the original model performance.

### 6.3 Task generalization

Finally, using the trained network from Section 6.1, we generate predictions for each of the three datasets (one for each pair of IMUs) generated from the three-finger hand (see Section 5). We use these predictions to generate an ROC curve for the network as applied to each dataset. We then consider the performance if these datasets were used together in an ensemble system where a consensus vote is used to produce a single prediction. That is, we use the model to produce a prediction for each pair of fingers, then assign a positive prediction only if the model predicts slip for all finger pairs.

## 7 Results & discussion

**FIGURE 10:**
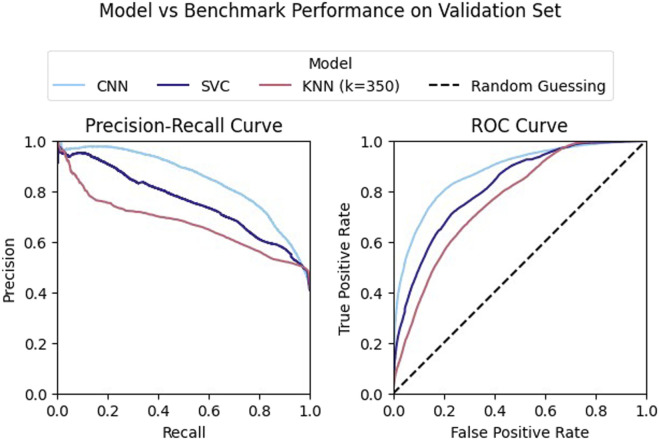
Our model outperforms the SVC and KNN algorithms on the validation dataset. All of the classifiers achieved a fit that performed better than chance.

**FIGURE 11:**
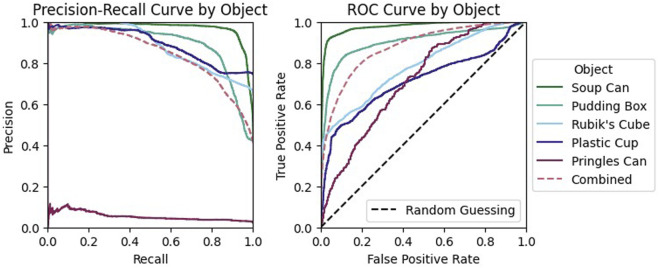
Slip detection by object: The network’s performance on the soup can or pudding box is noticeably better than for the other objects.

While our model had strong performance on the validation data Our model had strong performance on the validation data, outperforming both of the classical methods (see Figure 10). However, this performance varied greatly across objects. Performance was highest when classifying examples generated by grasping either the Jell-o pudding box or tomato soup can (Figure 11). Additionally, the model failed to achieve high precision when considering only examples generated by grasping the Pringles chips can. This is likely due to the very low percentage of positive examples in this subset of the data (see Table 3).

**FIGURE 12:**
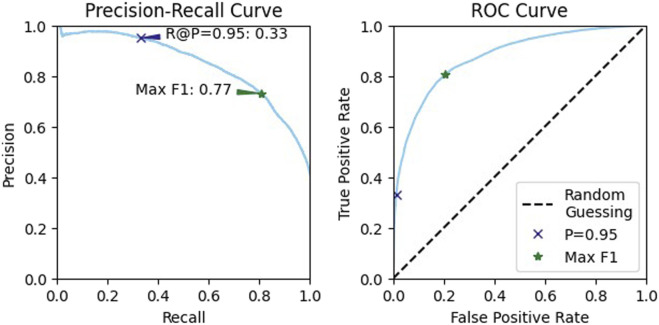
Precision recall curves and receiver operating characteristic (ROC) curves for the validation data. We used these to identify two thresholds for testing, one that maximizes the F1 score, for balanced performance, and one that achieves a high precision.

From the validation data, we selected a confidence threshold of 0.98 to represent the conservative, or high-precision, case and a threshold of 0.55 for balanced performance (Figure 12). Testing on our reserved data at these two thresholds resulted in a precision of 93.1% and a recall of 25.2% for the conservative case. For the balanced case, the precision was 64.0% and the recall was 77.2%.

**FIGURE 13:**
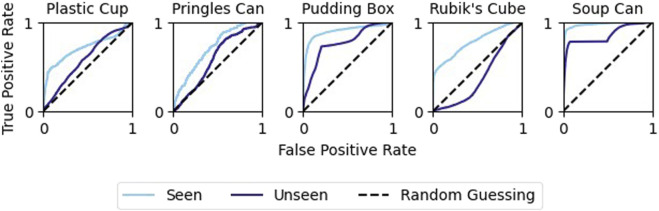
Object generalization: Comparing the predictive ability of the original network to the object-excluded one. As expected, the performance decreases, but is still better than random chance for all objects except the Rubik’s Cube.

**FIGURE 14:**
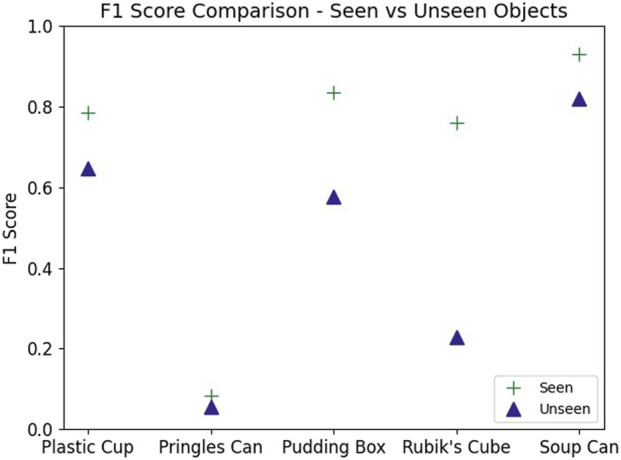
The maximum achievable F1 score decreases when validating the network on an object that the network has not been trained on. Performance remains high for the tomato soup can.

The ability of the model to generalize to unseen objects was mixed. The model performed better than chance for all unseen objects except the Rubik’s cube (Figure 13). However, in all cases, the model performed worse when predicting on unseen objects than on those that were present in the training data (Figure 14). Despite this, the model was still able to achieve an F1 score of 82.0% when the unseen object was the tomato soup can.

**FIGURE 15:**
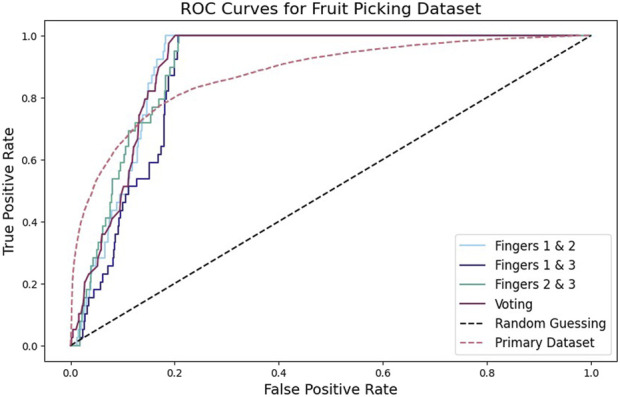
For the fruit picking dataset, we used a different inertial measurement unit, introduced a unique noise profile from robot motion, and used fingers that are arranged radially rather than in a flat plane. Yet, the network’s performance is similar to its performance on the original dataset.

The classifier also performed better than chance for nearly all confidence thresholds when tested on the fruit picking dataset (Figure 15). This was true for all three two-finger pairs, as well as for the voting system. Due to the low slip percentage (3.3%) in the dataset, the model struggled with precision. When the slip examples were upsampled to achieve class parity, an F1 score of 76.7% was achieved.

Our results show that for tendon-driven hands slip can be discriminated from environmental sliding using only fingertip IMUs. The overall performance when detecting slip using IMUs was similar to the performance reported in the literature for arrays of capacitive sensors (Heyneman and Cutkosky, 2016). One possible limitation to performance is that the sampling rate for the hand used in this study was only 40 Hz. This means that frequencies over 20 Hz could not be detected. It is also unclear how much of the predictive capability of the neural network is attributable to the correlation across sensor channels compared to the proprioceptive information captured by the gyroscope. Additional research is needed to determine the individual effects of vibration, fingertip deformation, and proprioception for robotic slip perception.

## 8 Conclusion

This paper supports the feasibility of fingertip-mounted IMUs, an increasingly common sensor configuration for compliant hands, to discriminate between slip and environmental sliding for such grippers. Our network was able to out-perform chance when generalizing to most unseen objects and when generalizing to an unseen dataset with a different gripper, object, and motion. However, the ability of the network to generalize to unseen objects was variable depending on the object in question. It is possible that the feature space for some sets of objects had a greater degree of overlap than for others. If this is the case, then feature engineering may be able to improve the generalizability.

Note also that in our primary dataset any amount of sliding between the hand and object is considered to be slip. In contrast, for many robotic manipulation tasks, only large, sustained, slip events that cause grasp loss are important. This is the type of slip labeled in our fruit-picking dataset. For such applications, the results presented here for the high-threshold case (precision = 93.1%, recall = 25.2%) may be sufficient for practical use.

## Data Availability

The datasets presented in this study can be found in online repositories. The names of the repository/repositories and accession number(s) can be found below: ”Slip Labels and Inertial Data from Compliant Hand (SLID-CH)” https://zenodo.org/records/15886336.
